# Construction and Analysis of Human Diseases and Metabolites Network

**DOI:** 10.3389/fbioe.2020.00398

**Published:** 2020-04-30

**Authors:** Kai Mi, Yanan Jiang, Jiaxin Chen, Dongxu Lv, Zhipeng Qian, Hui Sun, Desi Shang

**Affiliations:** ^1^College of Bioinformatics Science and Technology, Harbin Medical University, Harbin, China; ^2^Department of Pharmacology (State-Province Key Laboratories of Biomedicine – Pharmaceutics of China, Key Laboratory of Cardiovascular Research, Ministry of Education), College of Pharmacy, Harbin Medical University, Harbin, China; ^3^Translational Medicine Research and Cooperation Center of Northern China, Heilongjiang Academy of Medical Sciences, Harbin, China; ^4^School of Medical Informatics, Harbin Medical University, Daqing, China; ^5^Pharmaceutical Experiment Teaching Center, College of Pharmacy, Harbin Medical University, Harbin, China

**Keywords:** disease, metabolite, network random, correlation, functional modules

## Abstract

The relationship between aberrant metabolism and the initiation and progression of diseases has gained considerable attention in recent years. To gain insights into the global relationship between diseases and metabolites, here we constructed a human diseases-metabolites network (HDMN). Through analyses based on network biology, the metabolites associated with the same disorder tend to participate in the same metabolic pathway or cascade. In addition, the shortest distance between disease-related metabolites was shorter than that of all metabolites in the Kyoto Encyclopedia of Genes and Genomes (KEGG) metabolic network. Both disease and metabolite nodes in the HDMN displayed slight clustering phenomenon, resulting in functional modules. Furthermore, a significant positive correlation was observed between the degree of metabolites and the proportion of disease-related metabolites in the KEGG metabolic network. We also found that the average degree of disease metabolites is larger than that of all metabolites. Depicting a comprehensive characteristic of HDMN could provide great insights into understanding the global relationship between disease and metabolites.

## Introduction

The relationship between the environmental and genetic factors underlying various diseases is an important question in modern medicine (Autrup, 2005; Korbsrisate et al., 2007; Chanda et al., 2009; Pereyra et al., 2016). In recent years, genomics, proteomics, and metabolomics have provided new insights into monitoring disease progression, nutritional interventions and drug toxicities, and elucidated the causes of various diseases, and discovered potential links between seemingly different diseases (Eriksson et al., 2004; Pognan, 2004). Multiple-omics studies now indicate that pathological conditions are closely related to metabolic abnormalities (Griffin et al., 2002; Jarvela and Glueck, 2002; Gille et al., 2005; Chen and Hofestadt, 2006; Mombach et al., 2006). Complex diseases like cancer, diabetes, Alzheimer’s disease (AD), cardiovascular disease, schizophrenia, etc., are caused by the interactions between multiple genes and environmental factors. Consequently, exploring the metabolomes or metabolite profiles of these diseases have gained considerable attention in the post-genomic era (Yang et al., 2004; Mishur and Rea, 2012; Yu and Liang, 2012; Yu et al., 2017). Metabonomics is increasingly used in cancer biology to identify potential novel therapeutic targets. However, systematic metabonomic studies of cancer and other complex diseases are lacking. The systematic study of metabolites will help us to break the bottleneck of clinical treatment of complex diseases (Kim et al., 2009, 2011).

The metabolome of an organism or population reflects the genes, diet, lifestyle, and intestinal microbiota in that entity. In addition, the metabolic phenotype of an individual can be indicative of an abnormal biochemical or physiological state (Burgdorf et al., 2010; Reed et al., 2014; Colombo et al., 2018; Gar et al., 2018). Metabolic dysregulation is a major cause of various diseases, including diabetes, cardiovascular diseases, neuronal diseases, and cancers (Akinyemiju et al., 2017; Chong et al., 2017; Herholz et al., 2018). A pathological state can significantly alter metabolic pathways, resulting in aberrant levels of intermediates or end-products that can be viewed as potential diagnostic biomarkers or even therapeutic targets. However, few studies have analyzed metabolite levels and their functional relevance in diseases, which limits their potential in diagnosis or therapy (Krumsiek et al., 2012). Furthermore, the recent studies that have explored the dysregulated metabolic pathways in various diseases (Li et al., 2011) have also not analyzed the role of specific metabolites. Many complex diseases are accompanied by multiple metabolic processes and a metabolic process may be related to a variety of diseases. Therefore, it is essential to explore the global relationship between diseases and metabolomes in order to determine the role of metabolism in disease development and progression. The Human Metabolome Database (HMDB), which contains information of 625 human diseases and 110,000 metabolites (Wishart et al., 2018), is a helpful tool for studying the relationship between diseases and metabolomes.

To supplement the Kyoto Encyclopedia of Genes and Genomes (KEGG) program that identifies drug targets in metabolic pathways (Li et al., 2009). In this study, we constructed a human disease-metabolites network (HDMN) in which nodes represent diseases and metabolites and they were connected if there is association between disease and metabolites. By analyzing the topological properties of the network and mining functional modules, we investigated the internal mechanism of metabolite disorder in human body and provided an effective way for clinical research. Our results showed that the HDMN may not only offer insights into understanding underlying mechanisms of metabolic process but also provides a rational way to improve the interplay between metabolites and human diseases.

## Materials and Methods

### Human Metabolome Database

To construct the HDMN, the disease-metabolite correlations were first downloaded from the HMDB database. The HMDB is a up-to-date online metabolic database containing comprehensive information about human metabolites and their biological roles, physiological concentrations, pathological associations, chemical reactions, metabolic pathways, and reference spectra (Wishart et al., 2018). Next, we merged redundant terms and removed entries of predicted ones. In addition, the metabolites-pathway associations were obtained from the KEGG Pathway Database. The metabolites in each disease category was divided into 12 metabolic pathways, and each disease was grouped under a disease class. Finally, we obtained 28 disease classes for the 625 diseases in HMDB, along with 5475 unique disease-metabolites terms. Furthermore, in order to evaluate the role of disease metabolites in the global metabolic pathway, we reconstructed the KEGG metabolic network, in which 3617 metabolites and 4771 edges were obtained from the KEGG PATHWAY database.

### Distribution of Metabolites in Metabolic Pathways According to Disease Classification

The disease-related metabolites were first classified into 28 categories according to the disease class. After obtaining the relationship between disease classes and metabolic pathway categories based on shared metabolites, the distribution of metabolites of each metabolic pathway across the 28 diseases classes was calculated using the Hypergeometric test (*P*-value < 0.01).

### Disease-Metabolites Associations of HDMN

The shortest distance between any two metabolites in the KEGG metabolic network was calculated in reconstructing the metabolite-metabolite network using the metabolites-enzyme correlations (Yao et al., 2015). If two metabolites were in the same reaction, they were connected by one side. The shortest distance of node *i* and *j* was defined as:

short⁢distance⁢[i]⁢[j]=min⁡{[i],[j]} 

(0≤i≤n-1, 0≤j≤n-1)

where *i* and *j* are any two metabolites in the network.

### Definition of Disease Score

To determine whether the metabolites associated with the same disease are more likely to participate in the same metabolic reaction or pathway, we introduced a “disease score” (DS) defined as the maximum fraction of metabolites associated with a common disorder that are involved in a specific pathway. A metabolites-pathway matrix was first established for the metabolites in each disease, with rows for metabolites and columns for the metabolic pathways. If a metabolite belonged to a certain metabolic pathway, the corresponding cell was filled with 1, otherwise 0. The DS was calculated as follows:

$${\text{DS}}_{\text{k}}=\max\left\{\frac{\sum_{i=1}^{n}M_{i}^{P_{j}}}{n_{M}}\right\},i\,f\,\left\{ \begin{array}{c} M_{i}=1,M_{i}\in P_{j}\\ M_{i}=0,M_{i}\notin P_{j} \end{array}\right.$$

where DS*_*k*_* is disease k (625 diseases), *n*_*M*_ is the total number of metabolites in the disease, $M_{i}^{P_{j}}$ is the value of i metabolite in the *P*_*j*_ pathway (0 or 1). The diseases associated with only one metabolite were removed since the DS value of 1 for these diseases would affect the results. The significance is obtained by comparing the true distribution of DS with the randomized one in 10^3^ randomized networks generated by randomly shuffling the associations between metabolites and diseases while keeping the number of links per metabolite and disease unchanged.

### Degree Comparison of Disease Metabolites and All Metabolites

The average degree of disease metabolite and total metabolite nodes was calculated and

the average degree of nodes (*D*_*av*_) was defined as follows:

$$D_{a\,v}=\frac{2\,L}{N}$$

The degree of nodes (*N*_*d*_) was defined as follows:

$$N_{d}=E_{N_{i}}$$

where *N* is the number of nodes in the network, *L* is the number of edges in the network, and *E*_*N_i*_ is the number of edges directly connected to the *i* node in the network.

## Results

### Construction of the HDMN

**FIGURE 1:**
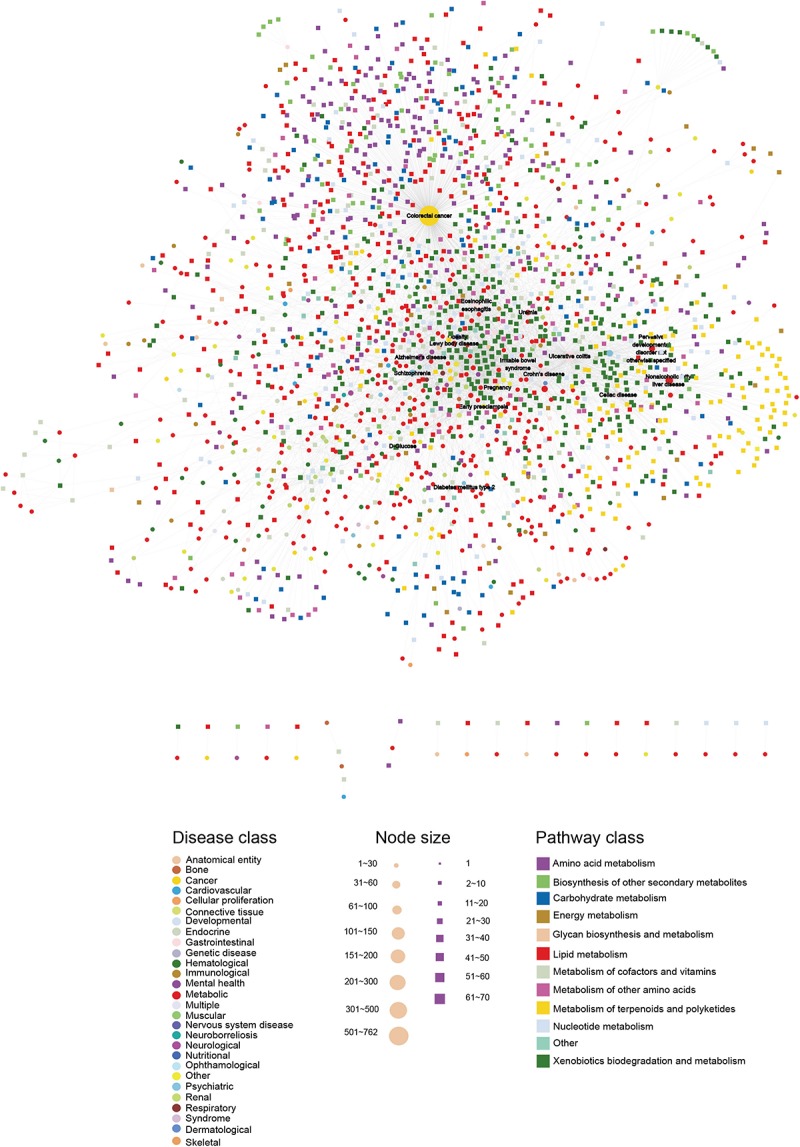
The HDMN network. The circles and rectangles in the network correspond to diseases and metabolites, respectively. The edges represent connections between a disease and a metabolite. The node size is proportional to its degree. The nodes are colored according to 28 disease classes and 12 KEGG pathway categories. The network has a total of 2339 nodes (625 disease nodes,1714 metabolite nodes) with 5475 edges.

To build the HDMN, we downloaded the diseases and metabolites data from the HMDB database, and merged redundant terms and removed entries of predicted ones. We obtained a total of 5475 unique disease-metabolites interactions consisting of 625 diseases and 1714 metabolites (Figure 1 and Supplementary Table S1). The diseases were grouped into 28 disease classes, and the metabolites into 12 metabolic categories. The metabolites of 408 diseases showed at least one link with the metabolites of another disease, indicating common genetic origins of most diseases.

### The Basic Network Features of the HDMN

**FIGURE 2:**
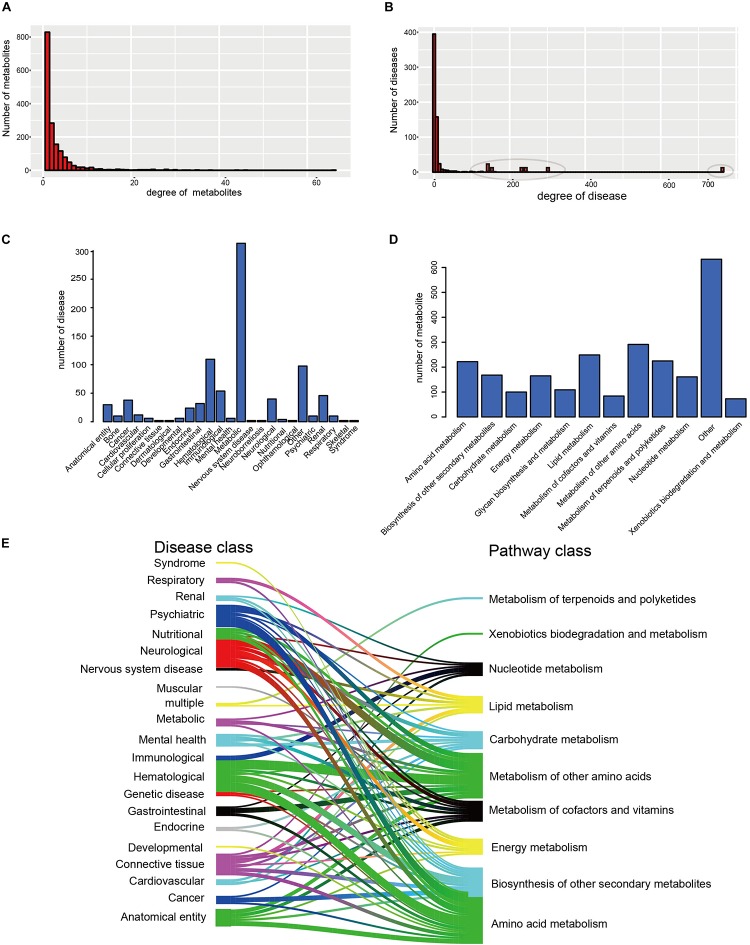
The basic network features of the HDMN. **(A)** Distribution of the number of mapped metabolites. **(B)** Distribution of the number of mapped diseases. **(C,D)** The distribution of nodes in the different disease and metabolic pathway categories. **(E)** The distribution of metabolites in each disease class in 12 metabolic pathways was calculated according to the hypergeometric test (*P*-value < 0.01).

Furthermore, the degrees of all disease nodes ranged from 1 to 762, while that of metabolites ranged from 1 to 66 (Figures 2A,B), indicating that few diseases are caused by aberrations in multiple metabolites. The degree of distribution of the node followed power law distributions (*R*^2^ = ∼0.781), thus confirming that the HDMN was scale-free (Xu et al., 2011). Disperse distribution of metabolite nodes suggest that some metabolites may play an important role to cause multiple disease; while some metabolites may serve as specific markers for few diseases.

We analyzed the distribution of nodes in the different disease and metabolic pathway categories in the HDMN, and found that diseases were concentrated in the metabolic diseases, gastrointestinal, Hematological, neurological and cancer categories (Figures 2C,D). We also found that lots of metabolites are belonging to lipid metabolism, amino acid metabolism and metabolism of other amino acids. To further investigate the relationships between disease and metabolite nodes of the HDMN, we next screened for the significantly overlapping metabolites between the 12 metabolic pathways and each disease class (Figure 2E), and found that the metabolites in five disease classes – hematological, anatomical entity, neurological, psychiatric, cancer – belong to the amino acid metabolism metabolic pathway. This is not surprising since any nutritional imbalance can affect development and hormonal functions (Guest and Guest, 2018). Furthermore, the metabolites of mental health, nervous system disease, cardiovascular and respiratory disease classes were linked to the lipid metabolism pathway. Metabolic myopathies cause exercise intolerance, myalgia, increased muscle breakdown products during exercise, as well as respiratory failure and obstructive sleep apnea (Bingol et al., 2018; Koo and Sethi, 2018).

### Cluster Analyses of the HDMN

**FIGURE 3:**
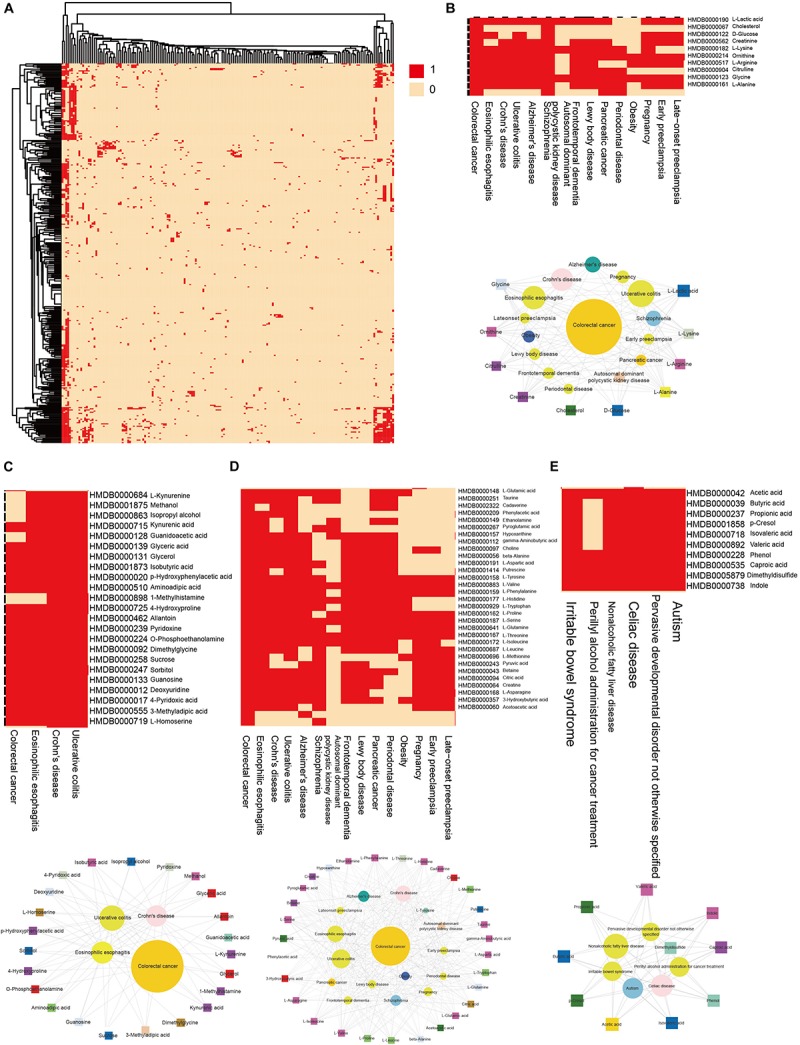
Hierarchical clustering on the HDMN and functional modules. **(A)** Hierarchical clustering of network hub nodes. The corresponding cell was colored red if there was an edge between the disease and metabolite in the HDMN. **(B–E)** Zoom-in plot showing some closely related functional modules.

We then clustering hub nodes (degree > 5) and identified the functional modules in the HDMN (Figure 3A). Hierarchical clustering of the HDMN indicated some closely related functional modules in the network, of which four modules were selected for further research (Figures 3B–E). For example, recent studies showed that the choline trimethylamine-lyase gene is overexpressed in colorectal cancer (CRC), indicating a relationship between microbiome choline metabolism and CRC (Thomas et al., 2019). Interestingly, three of these four functional modules include colorectal cancer, eosinophilic esophagitis, Crohn’s disease, and ulcerative colitis, although the metabolites were different among these modules.

### Global Propensity and Shortest Distance of Network

**FIGURE 4:**
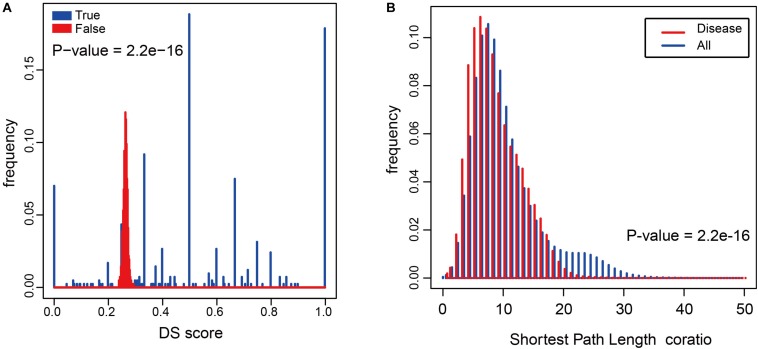
The relationship between diseases and metabolites in the HDMN. **(A)** Comparison of true DS score with random disturbance DS score (*P*-value = 2.2e-16, two sided Wilcox. Test). **(B)** The shortest distances between any two nodes in the metabolic network, and the shortest distance between disease metabolites (*P*-value = 2.2e-16, two sided Wilcox. Test).

To determine whether metabolites associated with the same disorder also participated in the same metabolic pathway or cascade, we generated a DS for each disease (see “Materials and Methods”) and based on their distribution. To evaluate its significance, we made 1000 randomly generated network of identical node and degree distribution for the disease-metabolite interaction association and carried out the same calculation steps for each disease to get the score of the disease. Concluded that metabolites linked to a disease tend to participate in one metabolic pathway (*P*-value = 2.2e-16, two sided Wilcox. Test, Figure 4A). Furthermore, the shortest distance between disease metabolites was shorter than that of all metabolites (*P*-value = 2.2e-16, two sided Wilcox. Test, Figure 4B), These results indicate that the metabolites are largely involved in the same metabolic reaction or adjacent reaction, and thus participate in cascade reactions. Taken together, metabolites that contribute to a common disorder have a tendency to interact with each other.

### Disease Metabolites Topological Analysis in Metabolic Network

**FIGURE 5:**
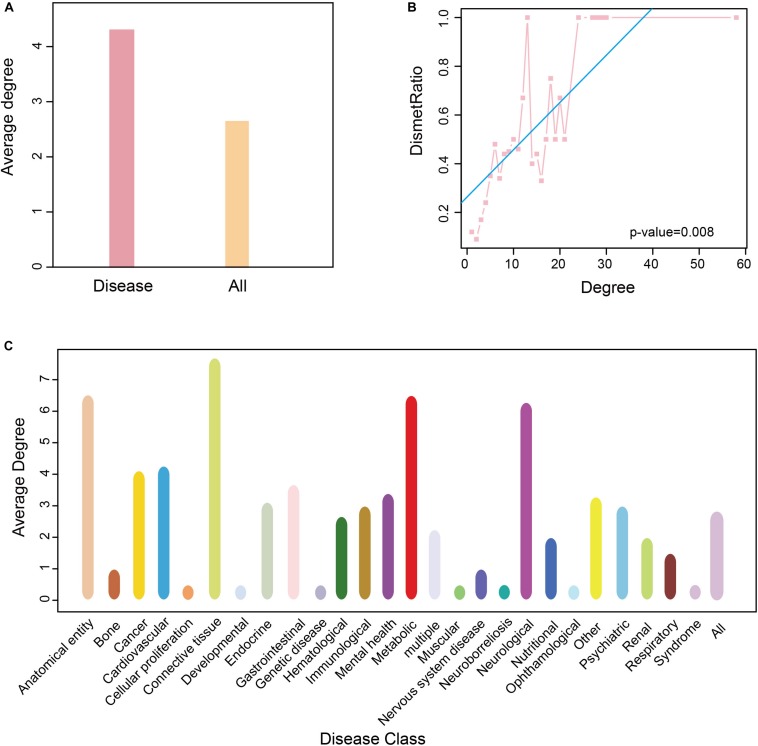
Degree of nodes association in HDMN. **(A)** The average degree of all metabolite and disease nodes. **(B)** The correlation between proportion of disease-related metabolites and degree (*P*-value = 0.008, *F*-test). **(C)** The average degree of metabolite in each disease class. The color of the disease corresponds to that of Figure 1.

To determine the role of these disease metabolites in metabolic networks, we calculated the average degree of metabolites in a reconstructed KEGG metabolic network (see “Materials and Methods”). The average degree of the disease metabolites was larger than that of all metabolites (Figure 5A), indicating that the former participates in more reactions. This raised the possibility that the more diseases a metabolite was related to, the higher degree it had in a metabolic network. Therefore, we calculated the proportion of disease metabolites in all metabolites under the same degree, and detected a positive correlation between the metabolite degree and the proportion of disease metabolites (*P*-value = 0.008, *F*-test, Figure 5B). Thus, metabolites associated with more diseases tend to participate in more reactions in metabolic networks. Finally, we calculated the average degree of each disease class and found that anatomical entity disease, connective tissue disease and neurological disease had higher average degree (degree > 5, Figure 5C).

## Discussion

Till date, studies conducted on the role of metabolites in diseases have mainly focused on the drugs and metabolic pathways (Li et al., 2011), the diseases and metabolic pathways (Li et al., 2012), or the metabolome in a single disease (Gonzalez et al., 2018; Xu et al., 2018; Che et al., 2019). The research on the relationship between diseases and metabolites is still limited, despite the fact that metabolic diseases have become highly frequent. To this end, we constructed a disease-metabolites network (HDMN) consisting of 2339 nodes (625 disease nodes and 1714 metabolite nodes) and 5475 edges, and reconstructed the metabolic network by extracting the relationship between metabolites and pathways from the KEGG database.

The distribution of the disease nodes was significantly broader than that of metabolite nodes in the newly constructed network, and the degree distribution of both obeyed the power law distribution. Disperse distribution of metabolite nodes suggest that some metabolites may play an important role to caused multiple disease, while some metabolites may serve as specific markers for few diseases (Figure 2A). Similarly, some metabolites were significantly linked to many diseases, indicating a common metabolic basis of these diseases (Figure 2B). However, there were only a few metabolites that specifically induced a certain disease. In recent years, studies have increasingly identified aberrant metabolites in complex metabolic diseases and cancers, which offers new possibilities of diagnosing and treating these disorders. The disease node with the greatest degree (degree = 762) was that of CRC, a major cause of morbidity and mortality worldwide. The feces of CRC patients show high levels of branched chain fatty acid (BCFA), isovalerate, isobutyrate, valerate, and phenylacetate, and low levels of amino acids, sugar, methanol, and bile acids (deoxycholate, stone deoxycholate, and cholate) (Le Gall et al., 2018; Shiao et al., 2018), indicating that dysregulated metabolites can increase the risk of intestinal cancer in humans. In addition, the degree of L-Lactic acid was the largest (degree = 66; Figure 2D) among the metabolite nodes, and thus likely dysregulated in multiple diseases. It participates in the xylose assimilation pathway. We found that most disease-related metabolites were mainly concentrated in some metabolic pathways, including lipid metabolism, amino acid metabolism and metabolism of other amino acids (Figure 2E). For example, amino acid metabolism was correlated with various diseases, including colorectal cancer, AD, and Crohn’s disease, etc. These relationships have been proved previously (Nakano et al., 2017; Le et al., 2018; Li et al., 2018). Some metabolites were highly disease-specific and associated with only one disease, such as arsenite, phenylalanine and lactulose. Arsenite exposure during development augments the severity of diet-induced fatty liver disease (Ditzel et al., 2016), patients with severe depression have different levels of phenylethylamine after overeating (Davis et al., 1994), and lactulose stimulates bowel movements as a disaccharide laxative and a prebiotic. It also modifies gut microbiota and ameliorates chronic kidney disease progression by suppressing uremic toxin production (Tayebi Khosroshahi et al., 2014; Tayebi-Khosroshahi et al., 2016; Sueyoshi et al., 2019). Taken together, these findings suggest that the occurrence of a disease is accompanied by local metabolic disorders.

Hierarchical clustering of network hub node further revealed several closely related functional modules, each with large degree interconnected nodes. To further explore the disease-metabolite associations, we classified the metabolites in each pathway according to the disease classes, and found that the metabolites significantly associated with a disease were more likely to participate in one metabolic pathway. To validate this conclusion, we built a 10^3^ randomly generated network of disease-metabolite interactions with identical node and degree distribution (Figure 4A, *P*-value = 2.2e-16, two sided Wilcox. Test), and found that the metabolites involved in each disease are closely related, or at least participate in the same reaction or cascade. Interestingly, the shortest distances between disease metabolites nodes were shorter and the average degree of disease metabolites was greater than that of all metabolites (Figure 4B, *P*-value = 2.2e-16, two sided Wilcox. Test), which further illustrates the close relationship between nodes in the HDMN. In addition, the proportion of disease-related metabolites in all metabolites increased significantly with the increasing degree (*P*-value = 0.008, *F*-test, Figure 5B), indicating that the greater the degree of nodes, the more complex the interaction between metabolites. Finally, the average degree of metabolites was higher in cancer, metabolic diseases, and neurological diseases (Figure 5C). Taken together, these results provided strong support for the functional importance of the HDMN. The HDMN also has clinical application significance in management of complex diseases. The clinical treatment of severe complex diseases was still a conundrum. Taken AD as an example, none of the current medication for AD has been shown to effectively reverse or even slow down its progression. Unfortunately, the approval rate of new AD drugs was significantly lower than in cancer and cardiovascular drugs. Whereas, HDMN could provide new ideas in the investigation and clinical treatment of this kind of diseases. In the HDMN, we observed a significant correlation between metabolic dysfunction and neurological diseases. Alzheimer’s disease and obesity are significantly correlated for they share many metabolites, including L-Asparagine, L-Aspartic acid, L-Isoleucine, and L-Serine. These metabolites belong to cyanoamino acid metabolism pathway. It has been proved that obesity significantly increase risk for AD (Profenno et al., 2010). The information in HDMN indicated that the restoration and maintenance of metabolic balance may be helpful in treating AD. The above findings indicated a promising prognostic and drug repurpose strategy for AD, as well as other complex diseases.

To summarize, we have effectively identified the intrinsic link between diseases and metabolites. The HDMN can identify key disease metabolites and provide new insights into the metabolic basis of complex disorders. Our future studies will focus on the closely linked functional modules and the metabolite nodes with greater impact and clinical relevance, as well as improving the quality of raw data to obtain a more accurate and robust network. The incompleteness of metabolite data, disease-metabolite associations and the false positive results greatly limited the completeness of the HDMN. With the development of clinical data and bioinformatics databases, this work will incorporate more data types. Although our data and methodology are far from completeness, our analysis of the HDMN, based on the network characteristics, still offers a comprehensive picture of global and significant associations between diseases and metabolites.

## Data Availability Statement

Publicly available datasets were analyzed in this study. This data can be found here: http://www.hmdb.ca.

## Author Contributions

DS and KM designed the study. KM, YJ, JC, DL, and ZQ collected the data. KM, JC, DL, and ZQ developed the computational model and analyzed the network. KM wrote the manuscript. All authors reviewed the manuscript.

## Conflict of Interest

The authors declare that the research was conducted in the absence of any commercial or financial relationships that could be construed as a potential conflict of interest.

## Supplementary Material

TABLE S1 Detail of the HDMN, including disease classification, KEGG ID and drug bank ID, etc.

## References

[B1] Akinyemiju T. Moore J. X. Judd S. Lakoski S. Goodman M. Safford M. M. (2017). Metabolic dysregulation and cancer mortality in a national cohort of blacks and whites. *BMC Cancer* 17:856. 10.1186/s12885-017-3807-2 29246121 PMC5731092

[B2] Autrup H. (2005). Genetic variations in the metabolism of environmental toxins. *Ugeskr. Laeger* 167 2173–2176. 15987077

[B3] Bingol Z. Tekce H. D. Sagcan G. Serdaroglu P. Kiyan E. (2018). Pulmonary functions and sleep-related breathing disorders in lipid storage disease. *Sleep Breath* 22 1137–1142. 10.1007/s11325-018-1647-1 29497949

[B4] Burgdorf K. S. Sandholt C. H. Sparso T. Andersen G. Witte D. R. Jorgensen T. (2010). Studies of association between LPIN1 variants and common metabolic phenotypes among 17,538 Danes. *Eur. J. Endocrinol.* 163 81–87. 10.1530/eje-10-0068 20356931

[B5] Chanda P. Sucheston L. Zhang A. Ramanathan M. (2009). The interaction index, a novel information-theoretic metric for prioritizing interacting genetic variations and environmental factors. *Eur. J. Hum. Genet.* 17 1274–1286. 10.1038/ejhg.2009.38 19293841 PMC2952438

[B6] Che J. Wang W. Huang Y. Zhang L. Zhao J. Zhang P. (2019). miR-20a inhibits hypoxia-induced autophagy by targeting ATG5/FIP200 in colorectal cancer. *Mol. Carcinog.* 58 1234–1247. 30883936 10.1002/mc.23006

[B7] Chen M. Hofestadt R. (2006). A medical bioinformatics approach for metabolic disorders: biomedical data prediction, modeling, and systematic analysis. *J. Biomed. Inform.* 39 147–159. 10.1016/j.jbi.2005.05.005 16023895

[B8] Chong C. R. Clarke K. Levelt E. (2017). Metabolic remodeling in diabetic cardiomyopathy. *Cardiovasc. Res.* 113 422–430. 28177068 10.1093/cvr/cvx018 PMC5412022

[B9] Colombo R. Damiani C. Gilbert D. Heiner M. Mauri G. Pescini D. (2018). Emerging ensembles of kinetic parameters to characterize observed metabolic phenotypes. *BMC Bioinformatics* 19:251. 10.1186/s12859-018-2181-7 30066662 PMC6201900

[B10] Davis B. A. Kennedy S. H. D’souza J. Durden D. A. Goldbloom D. S. Boulton A. A. (1994). Correlations of plasma and urinary phenylacetic acid and phenylethylamine concentrations with eating behavior and mood rating scores in brofaromine-treated women with bulimia nervosa. *J. Psychiatry Neurosci.* 19 282–288. 7918350 PMC1188609

[B11] Ditzel E. J. Li H. Foy C. E. Perrera A. B. Parker P. Renquist B. J. (2016). Altered hepatic transport by fetal arsenite exposure in diet-induced fatty liver disease. *J. Biochem. Mol. Toxicol.* 30 321–330. 10.1002/jbt.21796 26890134 PMC4940226

[B12] Eriksson L. Antti H. Gottfries J. Holmes E. Johansson E. Lindgren F. (2004). Using chemometrics for navigating in the large data sets of genomics, proteomics, and metabonomics (gpm). *Anal. Bioanal. Chem.* 380 419–429. 10.1007/s00216-004-2783-y 15448969

[B13] Gar C. Rottenkolber M. Sacco V. Moschko S. Banning F. Hesse N. (2018). Patterns of plasma glucagon dynamics do not match metabolic phenotypes in young women. *J. Clin. Endocrinol. Metab.* 103 972–982. 10.1210/jc.2017-02014 29244078

[B14] Gille C. Hoffmann S. Holzhutter H. G. (2005). Combining bioinformatics resources for the structural modelling of eukaryotic metabolic networks. *Genome Inform.* 16 223–232. 10.1093/bib/2.3.223 16362925

[B15] Gonzalez L. L. Garrie K. Turner M. D. (2018). Type 2 diabetes - An autoinflammatory disease driven by metabolic stress. *Biochim. Biophys. Acta Mol. Basis Dis.* 1864 3805–3823. 10.1016/j.bbadis.2018.08.034 30251697

[B16] Griffin J. L. Muller D. Woograsingh R. Jowatt V. Hindmarsh A. Nicholson J. K. (2002). Vitamin E deficiency and metabolic deficits in neuronal ceroid lipofuscinosis described by bioinformatics. *Physiol. Genomics* 11 195–203. 10.1152/physiolgenomics.00100.2002 12388797

[B17] Guest F. L. Guest P. C. (2018). Developmental origins of stress and psychiatric disorders. *Methods Mol. Biol.* 1735 47–58. 10.1007/978-1-4939-7614-0_3 29380306

[B18] Herholz K. Haense C. Gerhard A. Jones M. Anton-Rodriguez J. Segobin S. (2018). Metabolic regional and network changes in Alzheimer’s disease subtypes. *J. Cereb. Blood Flow Metab.* 38 1796–1806. 10.1177/0271678x17718436 28675110 PMC6168902

[B19] Jarvela I. Glueck S. B. (2002). Charting the effects of antioxidant therapy in the diseased brain: focus on “Vitamin E deficiency and metabolic deficits in neuronal ceroid lipofuscinosis described by bioinformatics”. *Physiol. Genomics* 11 183–184. 10.1152/physiolgenomics.00149.2002 12464692

[B20] Kim K. Aronov P. Zakharkin S. O. Anderson D. Perroud B. Thompson I. M. (2009). Urine metabolomics analysis for kidney cancer detection and biomarker discovery. *Mol. Cell. Proteomics* 8 558–570. 10.1074/mcp.m800165-mcp200 19008263 PMC2649817

[B21] Kim K. Taylor S. L. Ganti S. Guo L. Osier M. V. Weiss R. H. (2011). Urine metabolomic analysis identifies potential biomarkers and pathogenic pathways in kidney cancer. *OMICS* 15 293–303. 10.1089/omi.2010.0094 21348635 PMC3125558

[B22] Koo P. Sethi J. M. (2018). metabolic myopathies and the respiratory system. *Clin. Chest. Med.* 39 401–410. 10.1016/j.ccm.2018.02.001 29779598

[B23] Korbsrisate S. Trakulsomboon S. Damnin S. Gatedee J. Ratchtrachenchai O. A. Leelaporn A. (2007). Genetic variations in Aeromonas hydrophila isolates from clinical and environmental sources in Thailand. *Southeast Asian J. Trop. Med. Public Health* 38 721–727. 17883013

[B24] Krumsiek J. Suhre K. Evans A. M. Mitchell M. W. Mohney R. P. Milburn M. V. (2012). Mining the unknown: a systems approach to metabolite identification combining genetic and metabolic information. *PLoS Genet.* 8:e1003005. 10.1371/journal.pgen.1003005 23093944 PMC3475673

[B25] Le Y. Zhang S. Ni J. You Y. Luo K. Yu Y. (2018). Sorting nexin 10 controls mTOR activation through regulating amino-acid metabolism in colorectal cancer. *Cell Death Dis.* 9:666. 10.1038/s41419-018-0719-2 PMC5986761 29867114

[B26] Le Gall G. Guttula K. Kellingray L. Tett A. J. Ten Hoopen R. Kemsley K. E. (2018). Metabolite quantification of faecal extracts from colorectal cancer patients and healthy controls. *Oncotarget* 9 33278–33289. 30279959 10.18632/oncotarget.26022 PMC6161785

[B27] Li C. Li X. Miao Y. Wang Q. Jiang W. Xu C. (2009). SubpathwayMiner: a software package for flexible identification of pathways. *Nucleic Acids Res.* 37:e131. 10.1093/nar/gkp667 19706733 PMC2770656

[B28] Li C. Shang D. Wang Y. Li J. Han J. Wang S. (2012). Characterizing the network of drugs and their affected metabolic subpathways. *PLoS One* 7:e47326. 10.1371/journal.pone.0047326 23112813 PMC3480395

[B29] Li H. Ye D. Xie W. Hua F. Yang Y. Wu J. (2018). Defect of branched-chain amino acid metabolism promotes the development of Alzheimer’s disease by targeting the mTOR signaling. *Biosci. Rep.* 38:BSR20180127. 10.1042/BSR20180127 PMC6028749 29802157

[B30] Li X. Li C. Shang D. Li J. Han J. Miao Y. (2011). The implications of relationships between human diseases and metabolic subpathways. *PLoS One* 6:e21131. 10.1371/journal.pone.0021131 21695054 PMC3117879

[B31] Mishur R. J. Rea S. L. (2012). Applications of mass spectrometry to metabolomics and metabonomics: detection of biomarkers of aging and of age-related diseases. *Mass Spectrom. Rev.* 31 70–95. 10.1002/mas.20338 21538458

[B32] Mombach J. C. Lemke N. Da Silva N. M. Ferreira R. A. Isaia E. Barcellos C. K. (2006). Bioinformatics analysis of mycoplasma metabolism: important enzymes, metabolic similarities, and redundancy. *Comput. Biol. Med.* 36 542–552. 10.1016/j.compbiomed.2005.03.004 15913593

[B33] Nakano M. Tominaga K. Hoshino A. Sugaya T. Kanke K. Hiraishi H. (2017). Therapeutic efficacy of an elemental diet for patients with crohn’s disease and its association with amino acid metabolism. *Saudi J. Gastroenterol.* 23 20–27. 28139496 10.4103/1319-3767.199110 PMC5329972

[B34] Pereyra S. Bertoni B. Sapiro R. (2016). Interactions between environmental factors and maternal-fetal genetic variations: strategies to elucidate risks of preterm birth. *Eur. J. Obstet. Gynecol. Reprod. Biol.* 202 20–25. 10.1016/j.ejogrb.2016.04.030 27156152

[B35] Pognan F. (2004). Genomics, proteomics and metabonomics in toxicology: hopefully not ‘fashionomics’. *Pharmacogenomics* 5 879–893. 10.1517/14622416.5.7.879 15469409

[B36] Profenno L. A. Porsteinsson A. P. Faraone S. V. (2010). Meta-analysis of Alzheimer’s disease risk with obesity, diabetes, and related disorders. *Biol. Psychiatry* 67 505–512. 10.1016/j.biopsych.2009.02.013 19358976

[B37] Reed L. K. Lee K. Zhang Z. Rashid L. Poe A. Hsieh B. (2014). Systems genomics of metabolic phenotypes in wild-type Drosophila melanogaster. *Genetics* 197 781–793. 10.1534/genetics.114.163857 24671769 PMC4063932

[B38] Shiao S. P. K. Grayson J. Yu C. H. (2018). Gene-Metabolite interaction in the one carbon metabolism pathway: predictors of colorectal cancer in multi-ethnic families. *J. Pers. Med.* 8:26. 10.3390/jpm8030026 30082654 PMC6164460

[B39] Sueyoshi M. Fukunaga M. Mei M. Nakajima A. Tanaka G. Murase T. (2019). Effects of lactulose on renal function and gut microbiota in adenine-induced chronic kidney disease rats. *Clin. Exp. Nephrol.* 23 908–919. 10.1007/s10157-019-01727-4 30895529 PMC6555783

[B40] Tayebi Khosroshahi H. Habibzadeh A. Khoshbaten M. Rahbari B. Chaichi P. Badiee A. H. (2014). Lactulose for reduction of nitrogen products in patients with chronic kidney disease. *Iran J. Kidney Dis.* 8 377–381. 25194404

[B41] Tayebi-Khosroshahi H. Habibzadeh A. Niknafs B. Ghotaslou R. Yeganeh Sefidan F. Ghojazadeh M. (2016). The effect of lactulose supplementation on fecal microflora of patients with chronic kidney disease; a randomized clinical trial. *J. Renal. Inj. Prev.* 5 162–167. 10.15171/jrip.2016.34 27689115 PMC5040005

[B42] Thomas A. M. Manghi P. Asnicar F. Pasolli E. Armanini F. Zolfo M. (2019). Metagenomic analysis of colorectal cancer datasets identifies cross-cohort microbial diagnostic signatures and a link with choline degradation. *Nat. Med.* 25 667–678. 10.1038/s41591-019-0405-7 30936548 PMC9533319

[B43] Wishart D. S. Feunang Y. D. Marcu A. Guo A. C. Liang K. Vazquez-Fresno R. (2018). HMDB 4.0: the human metabolome database for 2018. *Nucleic Acids Res.* 46 D608–D617. 29140435 10.1093/nar/gkx1089 PMC5753273

[B44] Xu H. Wang Z. Zhu L. Sui Z. Bi W. Liu R. (2018). Targeted neurotransmitters profiling identifies metabolic signatures in rat brain by LC-MS/MS: application in insomnia, depression and Alzheimer’s disease. *Molecules* 23:2375. 10.3390/molecules23092375 30227663 PMC6225496

[B45] Xu J. Li C. X. Li Y. S. Lv J. Y. Ma Y. Shao T. T. (2011). MiRNA-miRNA synergistic network: construction via co-regulating functional modules and disease miRNA topological features. *Nucleic Acids Res.* 39 825–836. 10.1093/nar/gkq832 20929877 PMC3035454

[B46] Yang J. Xu G. Zheng Y. Kong H. Pang T. Lv S. (2004). Diagnosis of liver cancer using HPLC-based metabonomics avoiding false-positive result from hepatitis and hepatocirrhosis diseases. *J. Chromatogr. B Analyt. Technol. Biomed. Life Sci.* 813 59–65. 10.1016/j.jchromb.2004.09.032 15556516

[B47] Yao Q. Xu Y. Yang H. Shang D. Zhang C. Zhang Y. (2015). Global prioritization of disease candidate metabolites based on a multi-omics composite network. *Sci. Rep.* 5:17201. 10.1038/srep17201 PMC4657017 26598063

[B48] Yu M. Zhu Y. Cong Q. Wu C. (2017). Metabonomics research progress on liver diseases. *Can. J. Gastroenterol. Hepatol.* 2017:8467192. 10.1155/2017/8467192 PMC5339575 28321390

[B49] Yu Z. Liang F. R. (2012). Research advance in application of metabonomics in cardiovascular diseases. *Zhongguo Yi Xue Ke Xue Yuan Xue Bao* 34 413–417. 22954129 10.3881/j.issn.1000-503X.2012.04.020

